# *Hystrix Brachyura* Bezoar Characterization, Antioxidant Activity Screening, and Anticancer Activity on Melanoma Cells (A375): A Preliminary Study

**DOI:** 10.3390/antiox8020039

**Published:** 2019-02-12

**Authors:** Al’aina Yuhainis Firus Khan, Faizah Abdullah Asuhaimi, Tara K. Jalal, Fatimah Opeyemi Roheem, Hatim Abdullah Natto, Muhammad Farid Johan, Qamar Uddin Ahmed, Ridhwan Abdul Wahab

**Affiliations:** 1 Department of Biomedical Sciences, Kulliyyah of Allied Health Sciences, International Islamic University Malaysia, Kuantan Campus, Bandar Indera Mahkota, Pahang 25200, Malaysia; alainayuhainis@gmail.com (A.Y.F.K.); faizahaa@gmail.com (F.A.A.); tarajalal@ymail.com (T.K.J.); hanatto@uqu.edu.sa (H.A.N.); 2 Department of Pharmaceutical Chemistry, Kulliyyah of Pharmacy, International Islamic University Malaysia, Kuantan Campus, Bandar Indera Mahkota, Pahang 25200, Malaysia; bukolami_fatty@yahoo.com; 3 Department of Epidemiology, Faculty of Public Health and Health Informatics, Umm Al-Qura University, Makkah 24381, Saudi Arabia; 4 Department of Haematology, School of Medical Sciences, Universiti Sains Malaysia, Kubang Kerian, Kelantan 16150, Malaysia; faridjohan@usm.my

**Keywords:** porcupine bezoar, GCMS, antioxidant assay, apoptosis, traditional medicine

## Abstract

Porcupine bezoars (PBs) are masses of undigested calcareous concretions formed within the gastrointestinal tract. There are undocumented claims that PBs have antioxidant activity and can treat cancers. However, limited scientific study has been carried out to verify these traditional claims. Hence, this study was conducted to characterize the chemical profile and validate the antioxidant and anticancer activity against melanoma cells (A375). PB extract was initially subjected to Fourier-transform infrared spectroscopy (FTIR), gas chromatography–mass spectrometry (GCMS), total phenolic content (TPC), and total flavonoid content (TFC) analyses. The bioautography of antioxidant assays, namely 2,2′-azino-bis(3-ethylbenzothiazoline-6-sulphonic acid (ABTS), 2,2-diphenyl-1-picrylhydrazy (DPPH), and β-carotene was performed. An in vitro A375 cell viability assay, apoptosis assay, cell cycle arrest assay, and gene expression assay were carried out as well. The experimental finding revealed 5,10-diethoxy-2,3,7,8-tetrahydro-1H,6H-dipyrrolo[1,2-a:1′,2′-d]pyrazine, ursodeoxycholic acid, and cholest-5-en-3-ol (3 beta)-, carbonochloridate are major compounds detected in PB extract. PB extract has low phenolic content, viz. 698.7 ± 0.93 (µg GAE/5 mg dry weight). The bioautography antioxidant assays revealed a potent antioxidant effect (ABTS > DPPH > β-carotene), with free radical scavenging activity. Furthermore, PB extract exhibited dose- and time-dependent inhibition of cancer activity on A375 cells due to the exhibition of apoptosis via an intrinsic pathway.

## 1. Introduction

Cancer has been reported as the second leading cause of mortality worldwide, and is expected to surpass heart disease in the next few years [1]. One recent report has revealed that cancer incidence is predicted to increase to as many as 22 million cases per year [2]. Cancer is characterized by evasion of apoptosis or defective apoptosis mechanisms which in turn lead to uncontrollable development of cells [3]. Most anticancer drugs aim to halt cancer cells from further proliferating and metastasizing to other parts of the body. In spite of great advances in anticancer research, current chemotherapy drugs have been reported to leave cancer survivors with lifelong side effects [4,5]. Thus, investigation using a natural product with fewer side effects is crucial.

Antioxidants are the agents that have the ability to prevent, delay or eradicate oxidative stress of a molecule from atmospheric oxygen or reactive oxygen species [6,7]. Antioxidants play an important role in the prevention of various chronic diseases, such as cardiovascular disease, neuronal disease, cataracts, and several types of cancer via the pathophysiology mechanism known as oxidative stress. In this context, there is an increasing interest to search for natural antioxidants present in functional herbs, fruits, and vegetables. Phytoconstituents such as phenolics, carotenoids, anthocyanins, and tocopherol have been found to exert chemo-preventive and cardio-protective effects [8]. 

**Figure 1:**
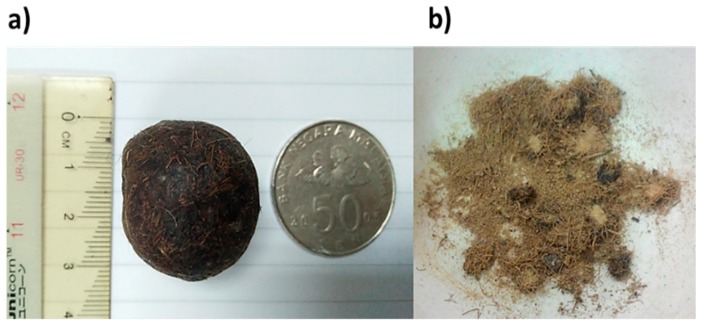
The porcupine bezoar (PB) used in this study. (**a**) PB with a Malaysian 50 sen coin and scale as size reference. (**b**) A PB after being crushed consists of a mixture of rough powder and undigested fiber.

In this study, the natural products used for antioxidant and anticancer evaluation is porcupine bezoar (PB) as depicted in Figure 1. PBs are concretions of undigested foreign materials that accumulate and calcify within the gastrointestinal tract of porcupines [9]. The bezoar is a stone-like material and is traditionally believed to exert various medicinal benefits [10,11]. The word bezoar itself has origins in Persian (pad = to expel, zahr = poison or antidote) [11]. Bezoar was valued as gem and as a noble metal. A review on the importance of bezoar has revealed that the oriental bezoars found in monkeys and porcupines (Lapis bezoar orientalis) were considered the most expensive [12,13,14]. Porcupine bezoars are generally composed of three concentric layers made up of vegetal matter [9]. Traditionally, people have used bezoars to treat several deadly diseases such as cholera, plague, smallpox, and measles, and as an antidote for poisons of different origins [7,8,9]. Due to traditional claims of medicinal benefits of porcupine bezoars and its rarity, traders tend to sell it at implausibly high prices. Moreover, there are undocumented traditional claims in several parts of Malaysia that porcupine bezoars can treat cancers and possess high antioxidant activity. Despite these undocumented traditional claims, there is limited information on the antioxidant and anticancer properties of porcupine bezoar. Therefore, the objective of this study was to characterize the PB chemical profile, investigate its antioxidant and anticancer properties to verify its traditional claim, and to explore its medicinal potential.

## 2. Materials and Methods 

### 2.1. Chemicals and Reagents

Methanol, Folin–Ciocalteu phenol reagent, and gallic acid were purchased from Honeywell (Charlotte, NC, USA). Quercetin, 1, 1-diphenyl-2-picrylhydrazyl radical (DPPH), trichloroacetic acid, and thin layer chromatography (TLC) plates were purchased from Sigma-Aldrich (St. Louis, MO, USA). Aluminium chloride hexahydrate (AlCl_3_·6H_2_O), sodium bicarbonate (Na_2_CO_3_), sodium phosphate dibasic (Na_2_HPO_4_), sodium phosphate monobasic (NaH_2_PO_4_), and ABTS [2,2′-azinobis (3-ethylbenzothiazoline)-6-sulfonic acid] diammonium salt were purchased from Roche, Basel, Switzerland. Human melanoma (A375) and normal human dermal fibroblast (NHDF) were obtained from American Type Culture Collection (ATCC; Manassas, VA, USA). Cells were grown in complete growth medium (CGM) which was made of Dulbecco’s modified Eagle medium (DMEM) (Nacalei Tesque, Kyoto, Japan) supplemented with 10% fetal bovine serum (Nacalei Tesque, Kyoto, Japan) and 1% penicillin–streptomycin (Nacalei Tesque, Kyoto, Japan). Phosphate-buffered saline (PBS) (Gibco, Waltham, MA, USA) was used for cell washing. For analyzing apoptosis and cell cycle, Nexin reagent (Merck Millipore, Burlington, MA, USA) and cell cycle reagent (Merck Millipore, Burlington, MA, USA) were used, respectively. For the mRNA expression analysis, InnuPREP DNA/RNA mini kit (Analytik Jena, Jena, Germany), SensiFAST cDNA synthesis kit (BIOLINE, Memphis, TN, USA), SensiFAST SYBR^®^ No-ROX Kit (BIOLINE, Memphis, TN, USA), and primer (Integrated DNA technologies, Singapore) were used. 5-Fluorouracil (5-FU) (Sigma-Aldrich, St. Louis, MO, USA), a standard anticancer drug, was used as a positive control.

### 2.2. Porcupine Bezoar Extract Preparation

The investigation using *Hystrix brachyura* bezoar was performed with the permission from the Malaysian government authority viz. Department of Wildlife and National Parks, Malaysia, for academic and research purposes (JPHL&TN (IP): 100-34/1.24 Jld 8). The PB (9.26 g) was crushed into a powdered form using mortar and pestle. Subsequently, 1 g of powdered PB was extracted using deionized water with 1:20 ratio for 30 min using ultrasonication method to get PB aqueous extract [15]. The supernatant was filtered and freeze-dried obtained PB aqueous extract in powdered form (250 mg, 25%). The dry powdered form of PB aqueous extract was then subjected to chemical profile characterization, bioautography antioxidant assay, and anticancer activity evaluation. For anticancer activity, PB aqueous extract was prepared into stock solution (2.0 mg/mL) and further diluted with CGM or DMEM accordingly. On the other hand, the positive control (5-FU) was prepared by dissolving in DMSO, before making it into stock solution (1.0 mg/mL) with CGM and diluted further accordingly. 

### 2.3. Fourier-Transform Infrared Spectroscopy Analysis Of Porcupine Bezoar

A Fourier-transform infrared (FTIR) spectrometer (Perkin Elmer Inc., Waltham, MA, USA) was used for the functional groups analysis. Prior to FTIR analysis, the PB aqueous extract was thoroughly freeze-dried to ensure an absence of water molecule. A small mass of PB aqueous extract was placed on the diamond crystal and the spectra were collected in wave number region of 400–4000 cm^−1^ at a resolution of 4 cm^−1^. The data were processed using PerkinElmer Spectrum (version 10.03.09) software (PerkinElmer, Waltham, MA, USA).

### 2.4. Gas Chromatography Mass Spectroscopy (GCMS) Profile Analysis of Porcupine Bezoar

The aim of this assay was to determine the chemical profile of PB aqueous extract. The PB aqueous extract was derivatized as described previously [16]. GCMS-TQ8030 linked to a GCMS-QP 2010 Plus Shimadzu gas chromatograph equipped with a capillary column DB-5 (0.25 μm thickness × 0.25 mm diameter × 30 mm length) was used for compound identification. Helium (1.0 mL/min) was used as the carrier gas (Shimadzu, Japan). The instrument was operated in electron impact mode at ionization voltage (70 eV), injector temperature (250 °C), and detector temperature (280 °C). The starting oven temperature was set at 50 °C and held for 5 min. This temperature was then programmed to 150 °C at 5 °C per min and held for 5 min. Finally, the temperature was programmed to 300 °C for 5 min at a rate of 5 °C per min. The compound identification from the spectral data was based on the available mass spectral records in National Institute of Standards and Technology database.

### 2.5. Total Phenol Content (TPC)

Folin–Ciocalteu’s method was modified and adapted to the 96-well plate assay as described by previously for the quantification of total phenolic content [17]. Mixtures of 25% (w/v) Folin–Ciocalteu’s reagent and 75% (w/v) sodium carbonate were prepared. Then, 50 μL of both mixtures were mixed with 10 μL of sample (5 mg/mL) and incubated for 45 min at room temperature. Absorbance was set at 765 nm using a Tecan microplate reader (Infinite^®^ 200 PRO, Männedorf, Switzerland) against blank. All assays were carried out at least in triplicate. The results were expressed as microgram gallic acid (GAE) equivalent per gram dry weight basis of the fresh sample (mg GAE/g dw basis).

### 2.6. Total Flavonoid Content (TFC)

Total flavonoid content was determined through modified method using 96-well plate as described earlier [17]. An aliquot of (10 μL) of sample (5 mg/mL) was mixed with 90 μL of 2% (w/v) aluminum chloride and incubated for 10 min at room temperature. The absorbance was set at 415 nm and the mixture was read using microplate reader (Tecan Infinite^®^200 PRO) against blank. All tests were performed in triplicate. The data were expressed as microgram quercetin (QE) equivalent per gram dry weight basis of fresh sample (mg QE/g dw basis).

### 2.7. Rapid Screening of Antioxidant by Dot-Blot Assay 

#### 2.7.1. DPPH Dot-Blot Assay

Sixteen grids of TLC plates were separately loaded with sample (PB aqueous extract) and standard (ascorbic acid) in decreasing concentrations (5–0.153 mg/mL) via two-fold serial dilution. The plate was left to completely dry at room temperature. Both plates were dipped for 10 s in 0.05% of DPPH in methanol. Excess solution was removed with tissue paper and the plate was air dried. After 30 min result was taken. Stained silica layer revealed a purple background with white/yellow spots at the location where radical-scavenger capacity presented. The intensity of the white/yellow color depends upon the amount and nature of radical scavenger present in the sample [18].

#### 2.7.2. ABTS^+^ Dot-Blot Assay

The 2,2′-azino-bis (3-ethylbenzothiazoline-6-sulfonic acid) diammonium salt radical cation (ABTS^+^) is a stable free radical frequently used for estimating the total antioxidant capacity of natural products. ABTS^+^ solution was prepared by mixing 7 mM ABTS stock solution with 2.45 mM potassium persulfate with ratio 1:1 (*v/v*) leaving the mixture in dark at room temperature for 16 h [19]. Dried TLC plates with pre-loaded sample (PB aqueous extract) and standard (ascorbic acid) were dipped in ABTS^+^ solution exhibited green background with white or pink spot indicated the presence of radical scavengers in PB aqueous extract [20].

#### 2.7.3. β-Carotene Dot-Blot Assay

Pre-loaded TLC plates with sample (PB aqueous extract) and standard (quercetin) were prepared and left to dry completely. The TLC plates were dipped in 0.05% solution of β-carotene in chloroform and exposed to sunlight for 2–3 hours or until the yellow background bleached [20]. Active compound will remain in yellow with white background.

### 2.8. Cell Culture Maintenance

Human malignant melanoma cells (A375) were maintained in complete growth medium (CGM) supplemented with 89% of Dulbecco’s modified eagle medium (DMEM), 10% fetal bovine serum and 1% of penicillin streptomycin antibiotic at 37 °C and 5% CO_2_ humidified atmosphere. 

### 2.9. Cytotoxicity Assay of Porcupine Bezoar on Cancer Cell and Normal Cell

This assay was performed to determine the median inhibition concentration on cancer cell of A375. Cell viability to determine 50% inhibitory concentration (IC_50_) and proliferation assay were evaluated using Promega CellTiter 96^®^ AQueous Non-Radioactive Cell Proliferation (Promega, Madison, WI, US) assay as recommended by the manufacturer. In determining IC_50_, cells were seeded at 4.0 × 10^2^ cells/mL and treated with PB extract with serial concentrations of 3.9–1000 µg/mL in a 96-well plate before incubation for 72 h. Then, 10 µL of MTS reagent were added in each well and incubated for 3 h before measuring at 490 nm using a microplate reader. The IC_50_ values for PB aqueous extract and 5-FU were calculated using Graphpad Prism 6. Further test for the proliferation assay over time (24, 48, 72 and 95 h) used untreated (UT) cells as negative control and 5-fluorouracil as positive control. Additionally, the IC_50_ of PB aqueous extract was evaluated on NHDF with UT and 5-FU as controls.

### 2.10. Cell Cycle Arrest Assay

Cell cycle assay was performed to determine whether PB aqueous extract has the ability to induce cell arrest in A375 cells and through which phase. Grown cells were treated with PB aqueous extract at IC_50_ for 72 h. All cells from the well were collected and prepared in triplicate. The cells were fixed with cold ethanol for 2 h and stained with cell cycle kit according to the manufacturer’s instructions. Cells were analyzed using guava flow cytometer (Luminex, Austin, TX, USA).

### 2.11. Apoptosis Assay

Apoptosis Annexin V/7AAD assay was performed to determine the apoptosis distribution. A375 cells were treated with PB aqueous extract at IC_50_ for 72 h. All cells from the well were harvested and prepared in triplicate. For apoptosis analyses, cells were stained using Nexin reagent and analyzed using guava flow cytometer following the manufacturer instruction. Distribution of early and late apoptotic cells after exposure with PB aqueous extract was reported in a dot plot graph.

### 2.12. Real Time Quantitative PCR (RT-qPCR) Analysis

The aim of this assay was to quantify the mRNA expression of mRNA of targeted primers related to apoptosis, cell cycle arrest and metastasis. Total RNA was extracted from PB aqueous extract-treated or untreated A375 cells after 72 h using RNA extraction kit according to the manufacturer’s instructions. The RNA’s quality and integrity were assessed before quantification process. The complementary DNA (cDNA) was prepared using cDNA synthesis kit according to manufacturer’s instructions. Subsequently, 200 ng of RNA template were synthesized into cDNA and later diluted in the experiments. The primers were chosen from the National Centre for Biotechnology Information (NCBI) database with a melting temperature (Tm) of 59–65 °C. Amplicon size was 70–150 bases. Forward and reverse primers spanning exon–exon junctions were selected to avoid amplification of genome sequences. PCR amplifications were performed using SYBR green in CFX96 Touch™ Real-Time PCR (Bio-Rad, Hercules, CA, USA). Annealing temperature was optimized using gradient temperature setting. Melting curves were analyzed to ensure amplification specificity and null primer-dimer formation. Primer PCR efficiency was evaluated using serial dilutions of cDNA sample (1:10, 1:100, 1:1,000, 1:10,000, and 1:100,000) in CFX Manager Software. The amplification efficiency (E) and correlation coefficients (R2) of the standard curve ranged from 93.0% to 114.7% and 0.984 to 0.998, respectively. Details information about the primers are attached in supplementary Table S1. The amplifications were done by following the conditions viz., 2 min at 50 °C, 10 min at 95 °C, and 45 cycles of 15 s at 95 °C and 1 min at 60 °C. Results were analyzed using CFX Manager Software based on the threshold cycle (CT) values. Glyceraldehyde 3-phosphate dehydrogenase (GAPDH) and β-actin (ACTB) were used as reference genes. The genes used, and the nucleotide sequences are summarized in Table 1.

### 2.13. Statistical Analysis

All values were presented as mean ± SD of triplicate from three different experiments (*n* = 3). A one-way analysis of variance (ANOVA) was performed using the Prism statistical software package (GraphPad Software, San Diego, CA, USA). This study used the Dunnet test as follow-up test of ANOVA which compared PB aqueous extract and 5-FU with UT. Differences between PB aqueous extract treated and UT were considered statistically significant at *p* < 0.01. 

## 3. Results

### 3.1. Porcupine Bezoar Characterization

**Figure 2:**
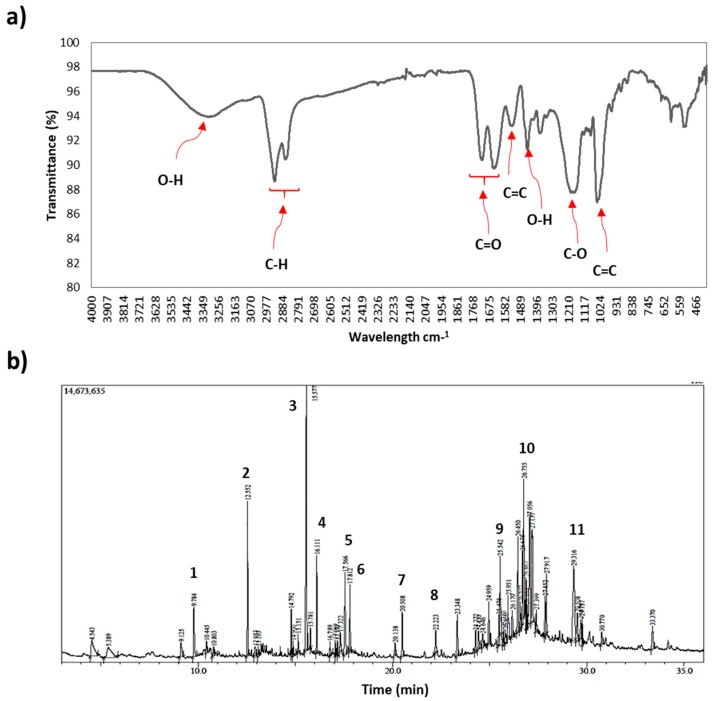
Porcupine bezoar chemical characterization. (**a**) The FTIR spectrum with its functional group detected. (**b**) The GCMS chromatogram of aqueous extract.

The chemical characteristic of the PB aqueous extract is presented in Figure 2a for FT-IR and Figure 2b for GCMS. The FT-IR spectrum depicted bonds relevant to hydrogen bonded O–H stretch vibrational bands at 3100 and 3690 cm^−1^ which might belong to carboxylic, alcohol, or phenol groups. Furthermore, a medium sp3 C–H stretch indicating an alkane group was determined at vibrational bands of 2800 and 3000 cm^−1^. Stretching weak alkene (C=C) and double medium carbonyl (C=O) were detected at vibrational bands of 1500–1580 cm^−1^ and 1580–1800 cm^−1^, respectively. Moreover, in the fingerprint region, a hydroxyl group was observed at 1400 cm^−1^, C–O stretching at 1200 cm^−1^, and C=C bending at 1050 cm^−1^.

Moreover, the PB extract was subjected to GC–MS analysis to evaluate PB extract chemical profile. The GC–MS retention time, % area peak, molecular formula and its similarity index are given in Table 2. The findings from Figure 2b and Table 2 show the major compounds detected are 5,10-diethoxy-2,3,7,8-tetrahydro-1H,6H-dipyrrolo [1, 2-a: 1′, 2′-d] pyrazine (11.28%), ursodeoxycholic acid (8.63%), cholest-5-en-3-ol (3.beta.)-, carbonochloridate (5.09%), and pentadecyl acrylate (4.10%). Details of each compound’s mass spectrum and structure are attached in supplementary Figure S1.

### 3.2. Total Phenolic Content and Total Flavonoid Content of Porcupine Bezoar

The total phenolic content (TPC) values were quantified based on the linear equation obtained from the gallic acid standard calibration curve. The TPC values were expressed as gallic acid equivalents (mg GAE/5 mg samples) at 698.7 ± 0.93. On the other hand, the total flavonoid content (TFC) was quantified based on the linear equation obtained from the quercetin standard calibration curve, and TFC values were expressed as the quercetin equivalent (mg QE/5 mg dry sample) which was −0.009 (mg/5 mg dry weight). The standard curves for TPC and TFC are attached in supplementary Figures S2 and S3.

**Figure 3:**
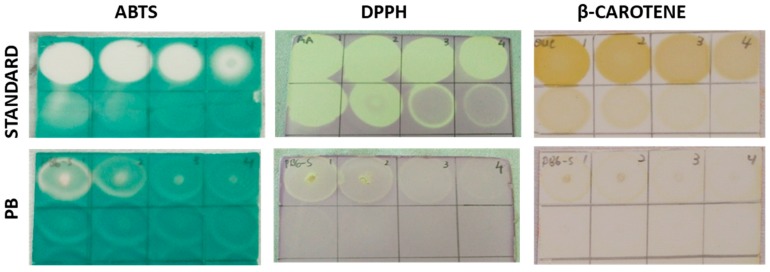
Dot-blot antioxidant of PB aqueous extract. Representative of dot-blot image of PB aqueous extract dilution series for ABTS, DPPH, and β-carotene bleaching antioxidants assay with ascorbic acid and quercetin as standard, respectively. For each assay, the initial concentration tested was 5.0 mg and further diluted in a 1:2 fold ration. (*n* = 3).

The aqueous PB extract was subjected to dot-blot antioxidant assay namely ABTS, DPPH and β-carotene. As depicted in Figure 3, for ABTS, the greenish area indicates no antioxidant activity while the bright white area indicates free radical scavenger. On the other hand, for DPPH the purple area on the plate indicates no free radical scavenging activity and the yellow area indicates antioxidant activity. As for the β-carotene assay, the brownish area indicates free radical scavenging of β-carotene. The dot-blot TLC plate showed the effect of PB aqueous extract on scavenging free radicals from serial concentration tested from 5.0 to 0.039 mg/mL. For the ABTS assay, the 5.0 mg/mL of PB extract showed to have similar dot-blot intensity with the fifth dilution (~0.3125 mg/mL) of the standard. For the DPPH assay, the highest concentration tested had similar dot-blot intensity with the seventh dilution (~0.078 mg/mL) of standard. On the other hand, β-carotene assay, the brownish intensity was similar with the eighth dilution (~0.039 mg/mL). The screening dot-blot antioxidant assay revealed PB aqueous extract to have ABTS > DPPH > β-carotene free radical scavenging activity.

### 3.3. Anticancer Activity of Porcupine Bezoar Aqueous Extract on Melanoma Cell (A375)

**Figure 4:**
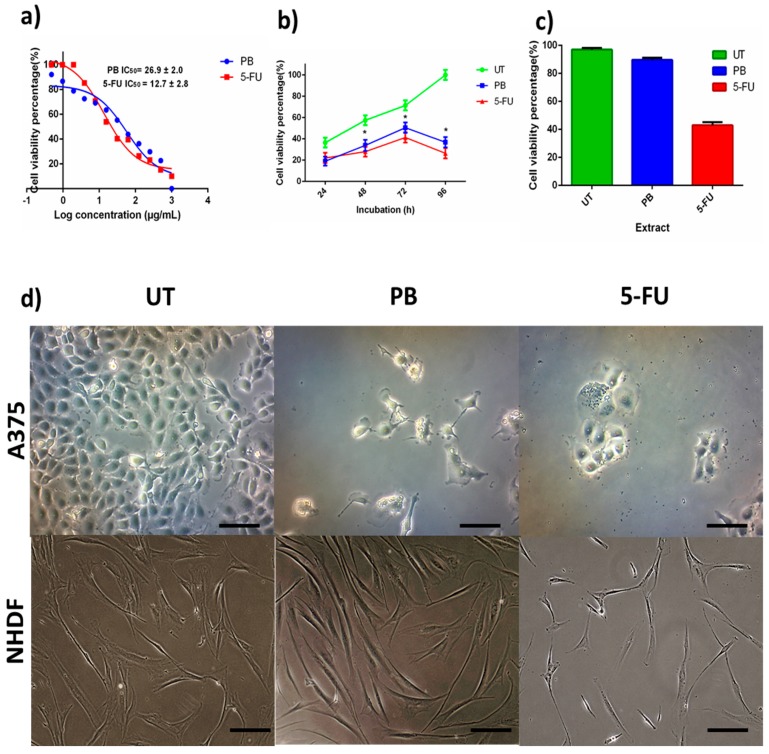
The effect of PB aqueous extract on A375 cells viability. (**a**) Median concentration of A375 treated with PB aqueous extract and 5-fluorouracil (5-FU). Graph shown are after the exposure with treatment for 72 h. IC_50_ is presented in mean ± SD (*n* = 3). (**b**) Anti-proliferative effect of PB aqueous extract at 24, 28, 72, and 96 h with untreated cells (UT) and 5-FU as controls. (**c**) Cytotoxic effect of median concentration on normal human dermal fibroblast (NHDF) cells at 72-h incubation. (**d**) Image of A375 and NHDF cells upon treatment with PB aqueous extract, UT, and 5-FU for 72 h. Bar graph = 25 µm. * indicates *p* < 0.01.

The cytotoxic effect of PB aqueous extract was evaluated on human melanoma, A375 cells. The A375 cells were subjected to various concentrations of PB aqueous extract for 72 hours and analyzed using MTS assay. The PB extract demonstrated a dose-dependent inhibitory effect on A375 cells growth. The IC_50_ values of PB are reported together with 5-FU in Figure 4a. The cytotoxic effect of IC_50_ of PB was further analyzed through proliferation assay. The result in Figure 4b demonstrated PB aqueous extract inhibits cell growth significantly across time incubation (24, 48, and 72 h) when compared to UT. Figure 4c presents the effect of IC_50_ of PB aqueous extract on NHDF. The graph revealed PB extract did not cause significant toxicity effect on cells viability as compared to 5-FU which caused significant cell viability reduction. Figure 4d shows that A375 cells treated with the PB aqueous extract demonstrated reduction of cells density compared to UT. Additionally, there were presence of cells debris in PB-treated cells. On the other hand, the untreated cells showed intact morphology of A375. Moreover, the morphology of NHDF supported the finding in Figure 4c as the morphology of PB aqueous extract-treated cells shows intact morphology of NHDF cells similarly to UT NHDF.

### 3.4. Porcupine Bezoar Aqueous Extract Induced Apoptosis on Melanoma Cell 

**Figure 5:**
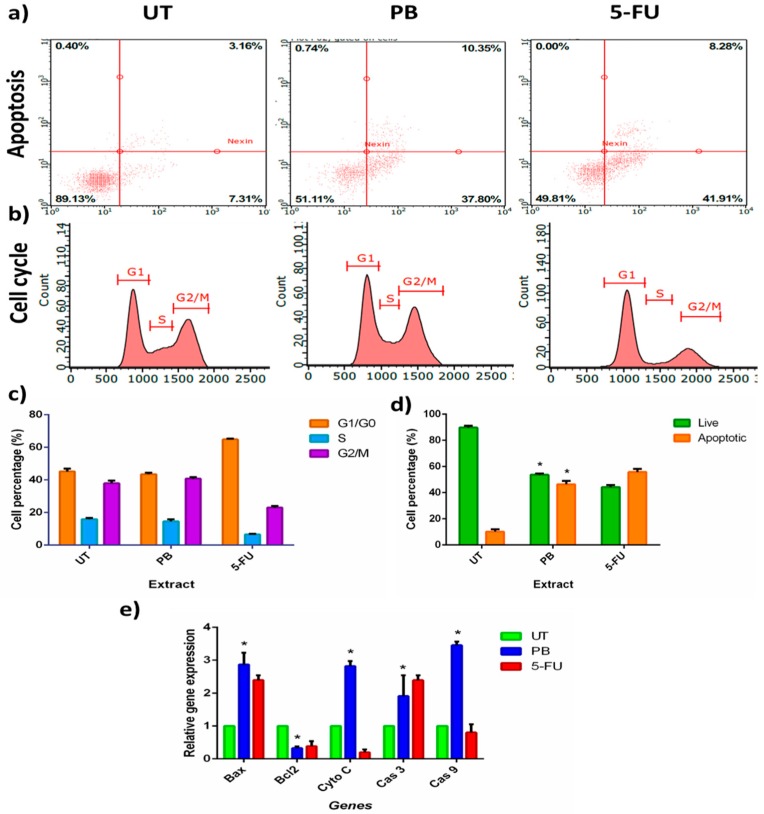
(**a**) Representation of DNA histogram of PB-treated cells, with UT and 5-FU as control. The analysis was after 72 h of incubation. DNA histograms displays cell cycle phases of treated cells, namely G1/G0, S, and G2/M (*n* = 3). (**b**) Representation of dot plot distribution of early and late apoptosis of A375 cells upon exposure with PB for 72 h with UT and 5-FU as control, (*n* = 3). (**c**) The proportion of cells according to cell cycle phase are presented in percentage mean ± SD (*n* = 3). (**d**) The proportion percentages of apoptotic (early and late apoptosis) and live cells are presented as percentage mean ± SD (*n* = 3). (**e**) The mRNA expression of apoptosis targeted genes is presented as relative gene expression mean ± SD (*n* = 3). The modulations of mRNA expressions levels of targeted genes are expressed as relative gene expression based on the calculation using GAPDH and β-actin as the reference gene, assigning the ratio in untreated cells as 1. * indicates *p* < 0.01.

Subsequently, the study explored the connection of A375 cell growth inhibition with cell cycle arrest. The PB aqueous extract treated A375 cells were exposed for 72 h and analyzed the cell cycle distribution using flow cytometry. Figure 5a,c, presents interesting findings as PB aqueous extract showed no evidence of inducing cells arrest in A375. The PB aqueous extract treated cells showed similar proportion of cells population with UT in all cell cycle phase. On the other hand, 5-FU treated cells showed cells arrest in G1/G0 phase where it can be seen that the cells accumulate in G1/G0 with reduction in S phase and G2/M phase.

Further analysis was carried out to evaluate whether PB aqueous extract inhibits the cells growth of A375 cells due to apoptotic activity. The PB aqueous extract treated cells were stained with Annexin V/7AAD. As presented in Figure 5b,d, PB aqueous extract induced apoptosis resulting in significant distribution of early apoptosis in PB aqueous extract treatment for 72 h. The dot-plot graph in Figure 5b demonstrates that PB aqueous extract induced early apoptosis by 37.80% and late apoptosis by 10.35% compared to UT, at 7.31% and 3.16%, respectively. Figure 5d reveals bar graph proportions of apoptotic cells (including early and the late apoptosis) and live cells which are 51.11% and 48.89%, respectively. To understand the mechanism of apoptosis at molecular level, qPCR experiment was performed on key genes viz., *Bax* (pro-apoptosis), *Bcl2* (anti-apoptosis), *cyto C*, *cas 3*, and *cas 9*. Figure 5e depicts that PB aqueous extract down-regulated *Bcl2* (0.3 fold), and up-regulated *Bax* (2.8 fold), *cyto C* (2.8 fold), *cas 3* (1.9 fold), and *cas 9* (3.4 fold). The mRNA expression finding indicated PB aqueous extract induced apoptosis via the mitochondria apoptosis pathway. As for 5-FU, it can be seen that apoptosis occurred, as *Bcl2* (0.4 fold) was down-regulated while *Bax* (2.4 fold), and *cas 3* (2.5 fold) were up-regulated. However, the pathway of 5-FU apoptosis could not be determined. 

## 4. Discussion

Porcupine bezoars have been used for past centuries for its medicinal benefits, however the information about its properties are still limited. Hence, the current study was performed to investigate the chemical characterization of PB, and evaluate its antioxidant activity and anticancer activity on melanoma cells. In the current study, the extraction method of PB was carried out using sonication procedure. As mentioned in [21], the sonication mechanism diffuses the solid area of the sample into the solvent. By forming cavitation bubbles that assist to break down the plant cells and increase the pores of the cell wall, the diffusion process is improved, leading to enhanced mass transfer into the solvent [22]. The time factor also is very important when using the sonication technique to extract the phenolic compounds. A longer extraction time allows more contact time for the cavitation bubbles to break more plant cells, resulting an increase of the extracted of phenolic compounds [23]. 

Phenolic compounds are powerful antioxidants and act in a structure-dependent manner; they can scavenge reactive oxygen species (ROS) and chelate transition metals which play vital roles in the initiation of deleterious free radical reactions [24]. Clearly, the total phenolic content is considered as an important indication of antioxidant properties of natural product extracts. Crude extracts of fruits, vegetables, and other natural product materials are rich in phenolic content. TFC was recorded as having the lowest antioxidant activity and the reason is due to the solvent chosen; this is in agreement with a previous study which reported that antioxidant activity of flavonoids depends on the structure and substitution pattern of hydroxyl groups [8]. Porcupines are herbivores and their dietary intake is from tree barks, seeds, twigs, and grasses [25,26]. This explains the reason for PB to have a low flavonoid content, as mostly flavonoid content can be found mainly in fruits, vegetables, leaves and is rarely found in bark or twigs [27]. Some of our initial data using different PB revealed that PBs have similar finding in this study where the phenolics content is low and absence of flavonoid content [28]. However, another study using three different types of PB (black, powdered, and grassy) reported black and powdered PB have significant higher amount of phenolic content compared to grassy PB [29]. Additionally, the GCMS profile also revealed main constituents are amino acids found in animal, which further clarified why PB extract have low phenolic and flavonoid content.

Food antioxidants play an important role in the promotion and maintenance of health as the antioxidant capacity is able to reduce the risk for chronic diseases such as cancer and heart disease [30]. ABTS antioxidant assay observed reaction kinetics of specific enzymes to act as electron donor to form the ABTS•+. Meanwhile, DPPH is a stable free radical and it receives an electron or hydrogen radical to become a stable diamagnetic molecule which is widely used to investigate radical scavenging activity. The ABTS and DPPH assays show PB aqueous extract had strong hydrogen-donating capacity. The results are in agreement with the phenol contents determined for each sample. Antioxidant agents act as reducing agents and antioxidants by the hydrogen-donating property of their hydroxyl groups [31]. The method is influenced by the solvent and the pH of the reactions. 

Therefore, it can be concluded here that the extraction of plants is related to its polarity of extraction solvent used. A previous study has reported that pentadecyl acrylate has DPPH and ABTS antioxidant activity [32]. Another study described ursodeoxycholic acid as having high capacity to scavenge hydroxyl and lipid peroxidation antioxidant assays [33]. Another study conducted antioxidant evaluation using three different types of bezoar namely black, grassy and powdered PB revealed that all three types of PB have different antioxidant capacity in 2, 2-diphenyl-1-picrylhydrazyl free radical scavenging activity assay (DPPH), ferric reducing power assay (FRAP), intracellular reactive oxygen species scavenging activity assay (ROS), and reactive nitric oxide scavenging activity assay [29]. However, the grassy PB displayed the lowest antioxidant activity. 

The cytotoxic assay revealed low concentrations for PB aqueous extract to inhibit 50% of A375 cell growth at 72 h. The cytotoxic study also suggested that PB exhibited cytotoxic and cytostatic capacity in a dose- and time-dependent manner. However, the flow cytometry analysis revealed PB did not cause cell cycle arrest but exhibited apoptosis. Annexin V/7AAD assay evaluated the cells and categorized them into a quadrant base in the presence or absence of phosphatidylserine and 7-AAD, which helped in differentiating early, late apoptotic and necrotic cells [34]. The PB aqueous extract was revealed to induce apoptosis when compared to UT cells and 5-FU treated cells. The underlying mechanism of apoptosis induction revealed PB aqueous extract exhibited apoptosis via mitochondria apoptosis pathway. The intrinsic pathway is initiated within cells involving the Bcl2 families such as pro-apoptosis (*Bax*) and anti-apoptosis (*Bcl2*) genes to regulate the promotion or inhibition of apoptosis [35,36]. PB aqueous extract also found to up-regulate the expressions of *cytochrome C*, *caspase 9*, and *caspase 3*. The finding was found to be in line with the intrinsic pathway in which internal stimuli induce bax to activate cytochrome C release into cytosol and activate caspase cascade through caspase 9 and caspase 3 [37,38]. The GCMS analysis revealed two compounds detected from PB aqueous extract possessed anticancer activity, namely stearic acid and ursodeoxycholic acid. Stearic acid is an 18-carbon chain of fatty acid found primarily in animal derivatives and plant fat [39]. Stearic acid has earlier been reported to induce apoptosis in MDA-MB-231 through mitochondria pathway by reducing mitochondria membrane potential, which leads to the release of cytochrome c into cytosol [40,41]. Ursodeoxycholic acid described to exhibit anticancer properties by inhibiting cell proliferation through regulation of oxidative stress in colon cancer cells [42]. However, remaining compounds are yet to be explored for their biological potential.

The bezoars are formed from the reaction of undigested porcupine food and enzymes from porcupine itself. The bezoar itself is a medical illness (gastrolith) for porcupines and needs to be surgically removed for the porcupine to survive. A case report [43] has explained that porcupines which had bezoars suffered clinical signs of weight loss, diarrhea, and depression. Ignoring the condition leads to death as the bezoar causes gastric perforation. Additionally, it is crucial to understand that each PB is unique in each porcupine. The chemical profile of each PB depends on the food habits of individual porcupine; the age of the bezoar in the porcupine and the location of the bezoar collected could also play major roles in the different chemical profiles of the bezoar.

## 5. Conclusions

The findings in this study have reported PB aqueous extract’s chemical profile, antioxidant activity, and anticancer activity on A375 cells for the first time. PB aqueous extract revealed 5,10-diethoxy-2,3,7,8-tetrahydro-1H,6H-dipyrrolo [1, 2-a: 1′, 2′-d] pyrazine, ursodeoxycholic acid, cholest-5-en-3-ol (3.beta.)-, carbonochloridate, and pentadecyl acrylate as the major constituents. The compounds that may be responsible for the anticancer activity of PB aqueous extract are ursodeoxycholic acid and stearic acid. However, other unidentified compounds which were not present in the library of the GC-MS system may also play some roles towards its antioxidant and anticancer effects. The PB showed low TPC and TFC values, which play major roles in the antioxidant activity (ABTS > DPPH > β-carotene). PB extract was shown to inhibit cell growth through induction of apoptosis via the mitochondria pathway with minimal toxicity to NHDF cells. However, further study is still necessary to isolate active compounds of PB and evaluate their antioxidant and anticancer properties along with safety profile.

## Figures and Tables

**Table 1:** Primer sequences used in quantitative polymerase chain reaction analysis.

Primer	Forward Sequence 5′ to 3′	Reverse Sequence 5′ to 3′
ACTB	F-CGGCGCCCTATAAAACCCA	R-ATCATCCATGGTGAGCTGGC
GAPDH	F-GACAGTCAGCCGCATCTTCT	R-GCGCCCAATACGACCAAATC
BAX	F-GAACCATCATGGGCTGGACAT	R-ATGGTCACGGTCCAACCACC
BCL2	F-ATGTGTGTGGAGAGCGTCAA	R-GGGCCGTACAGTTCCACAAA
CYTOCHROME C	F-CCCAAGAAGTACATCCCTGGAAC	R-GGCAGTGGCCAATTATTACTCA
CAS 3	F-TGGTTTGAGCCTGAGCAGAG	R-TGGCAGCATCATCCACACAT
CAS 9	F-TGACCCCAGAATTGACCCTG	R-AAGGATTCGCTCTTGCGTC

**Table 2:** GC-MS profile analysis of PB aqueous extract.

Peak	Retention Time	Area (%)	Molecular Formula	Compound	Similarity Index
1	9.784	2.91	C_12_H_26_O	1-Dodecanol	97
2	12.552	4.10	C_18_H_34_O_2_	Pentadecyl acrylate	91
3	15.577	11.28	C_14_H_22_N_2_O_2_	5,10-Diethoxy-2,3,7,8-tetrahydro-1H,6H-dipyrrolo [1, 2-a: 1′, 2′-d] pyrazine	81
4	16.11	2.32	C_15_H_30_O_2_S	Lauryl 3-mercaptopropionate	93
5	17.566	3.50	C_18_H_36_O_2_	Stearic acid	93
6	17.812	2.00	C_16_H_33_NO	Palmitamide	94
7	20.508	2.12	C_18_H_37_NO	Octadecanamide	93
8	22.223	1.04	C_19_H_38_O_4_	Glyceryl 2-palmitate	81
9	25.542	1.63	C_21_H_42_O_4_	Octadecanoic acid, 2,3-dihydroxypropyl ester	82
10	26.755	5.09	C_28_H_45_ClO_2_	Cholest-5-en-3-ol (3 beta)-, carbonochloridate	81
11	29.316	8.63	C_24_H_40_O_4_	Ursodeoxycholic acid	82

## Supplementary Materials

The following are available online at http://www.mdpi.com/2076-3921/8/2/39/s1. Table S1: Primers for gene expression; Figure S1: Structure and mass spectrum of components of porcupine bezoar extract; Figure S2: Standard curve total phenolic content of gallic acid; Figure S3: Standard curve total flavonoid content of quercetin.
