# Supplementary material for: Hystrix Brachyura Bezoar Characterization, Antioxidant Activity Screening, and Anticancer Activity on Melanoma Cells (A375): A Preliminary Study
Source: Antioxidants (Basel). 2019 Feb 12;8(2):39. doi: 10.3390/antiox8020039 (PMC6406421; doi:10.3390/antiox8020039)

Table A : Primers for gene expression. The table include information forward and reverse gene sequence, the amplicon size, Efficiency value and the slope value from standard curve graph.

Primer	Forward sequence 5' to 3'	Reverse sequence 5' to 3'	Length (bp)	Efficiency (%)	Slope R^2
ACTB	CGGCGCCCTATAAAACCCA	ATCATCCATGGTGAGCTGGC	112	108.8	0.997
GAPDH	GACAGTCAGCCGCATCTTCT	GCGCCCAATACGACCAAATC	104	103.7	0.964
BAX	GAACCATCATGGGCTGGACAT	ATGGTCACGGTCCAACCACC	85	105.0	0.998
BCL2	ATGTGTGTGGAGAGCGTCAA	GGGCCGTACAGTTCCACAAA	143	111.5	0.990
CYTOCHROME C	CCCAAGAAGTACATCCCTGGAAC	GGCAGTGGCCAATTATTACTCA	120	114.7	0.997
CAS 3	TGGTTTGAGCCTGAGCAGAG	TGGCAGCATCATCCACACAT	122	106.5	0.984
CAS 9	TGACCCCAGAATTGACCCTG	AAGGATTCGCTCTTGCGTC	81	96.1	0.986

Figure A1 : Structure and Mass Spectrum of Components Porcupine Bezoar Extract

1. 1-Dodecanol

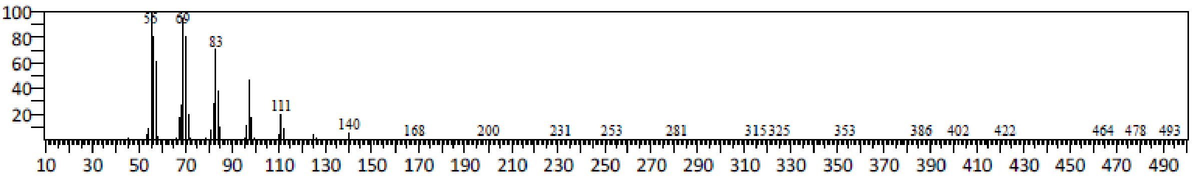

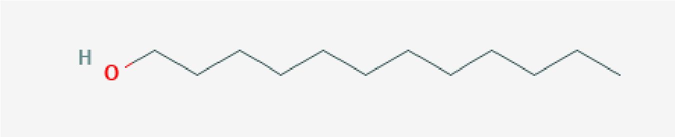

2. Pentadecyl acrylate

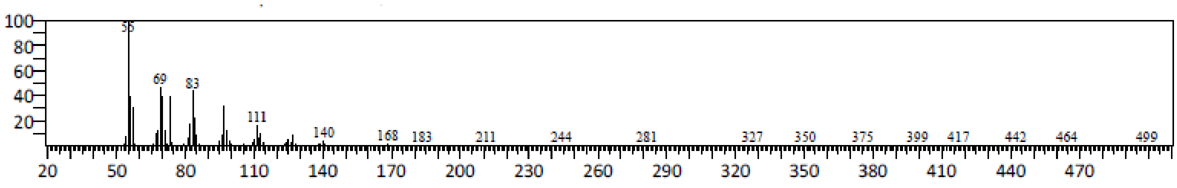

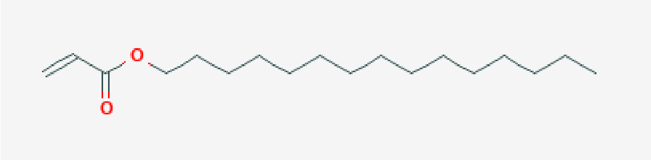

3. 5, 10-Diethoxy - 2, 3, 7, 8- tetrahydro -1H, 6H - dipyrrolo [1, 2-a: 1', 2'-d] pyrazine

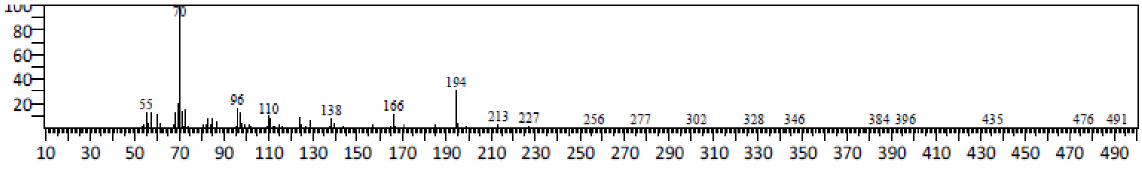

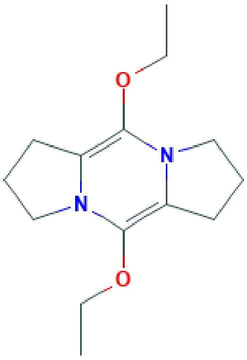

4. Lauryl 3-mercaptopropionate

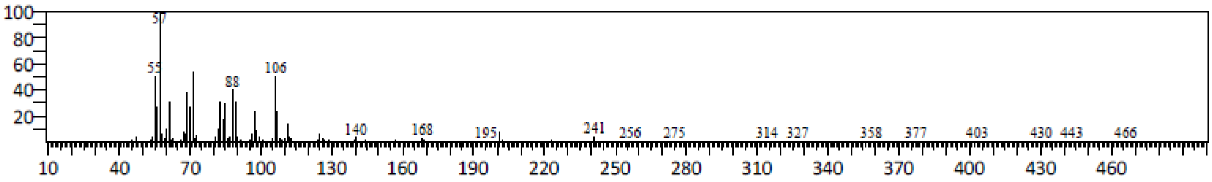

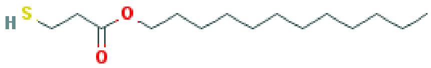

5. Stearic acid

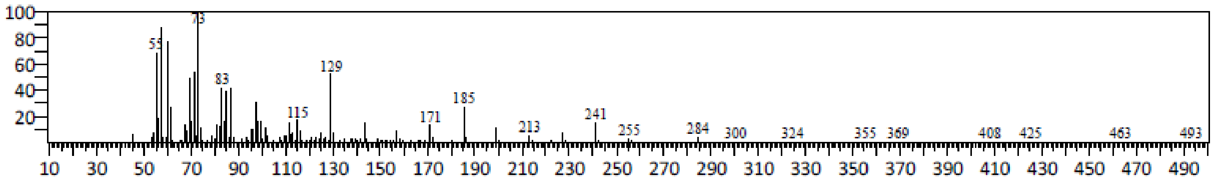

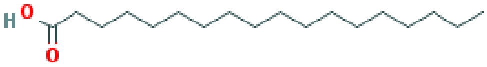

6. Palmitamide

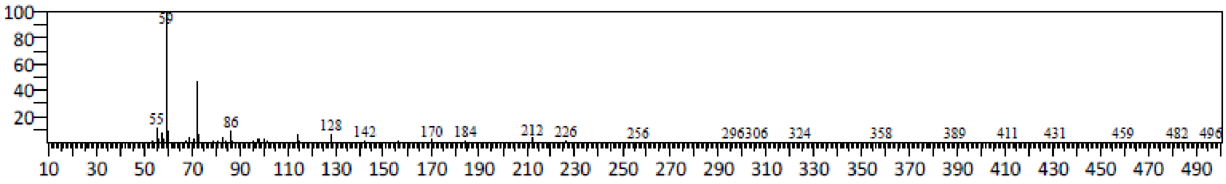

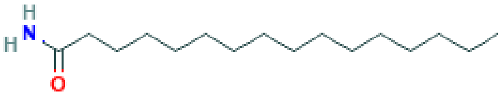

7. Octadecanamide

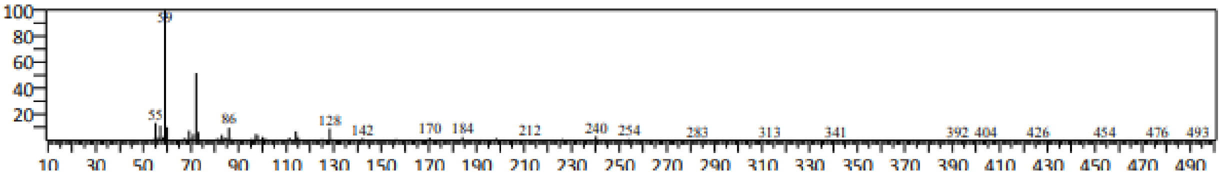

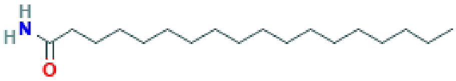

8. Glyceryl 2-palmitate

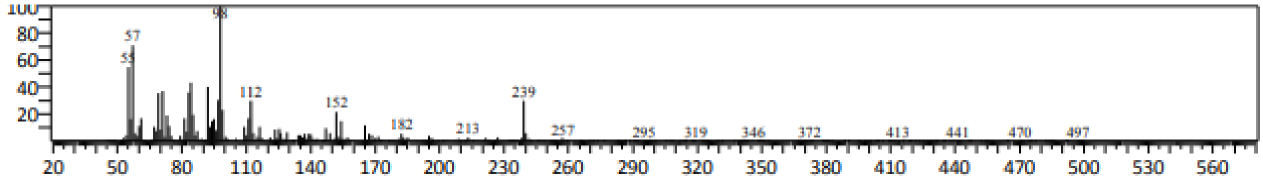

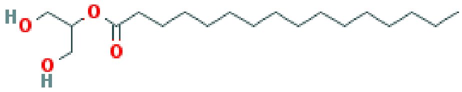

9. Octadecanoic acid, 2,3-dihydroxypropyl ester

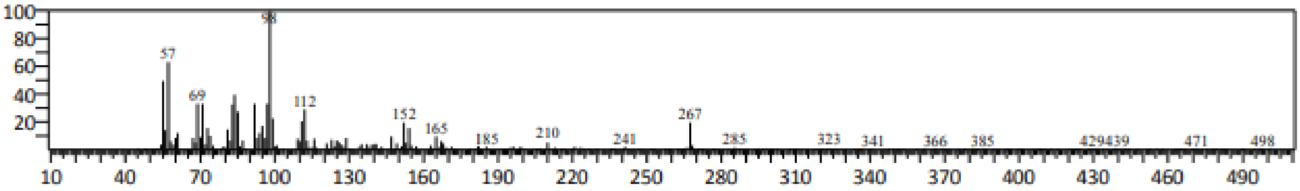

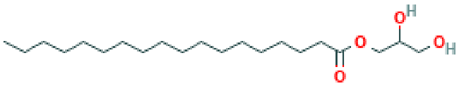

10. Cholest-5-en-3-ol (3.beta.)-, carbonochloridate

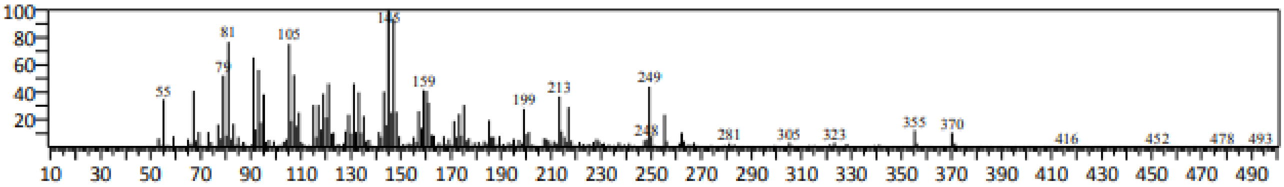

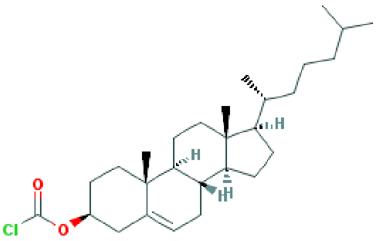

11. Ursodeoxycholic acid

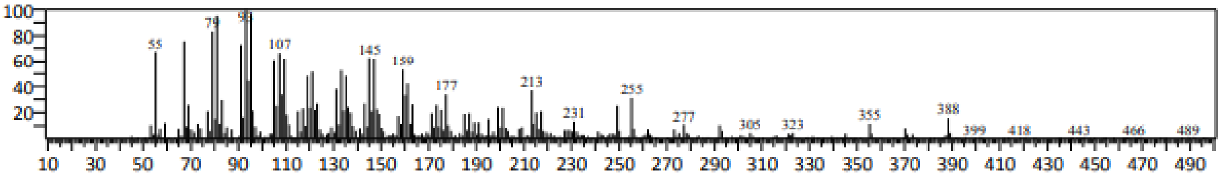

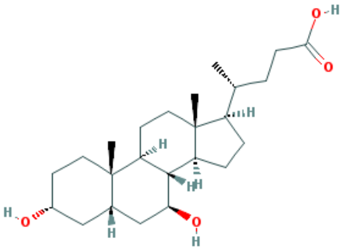

Figure A2 : Standard Curve Total Phenolic Content of Gallic Acid

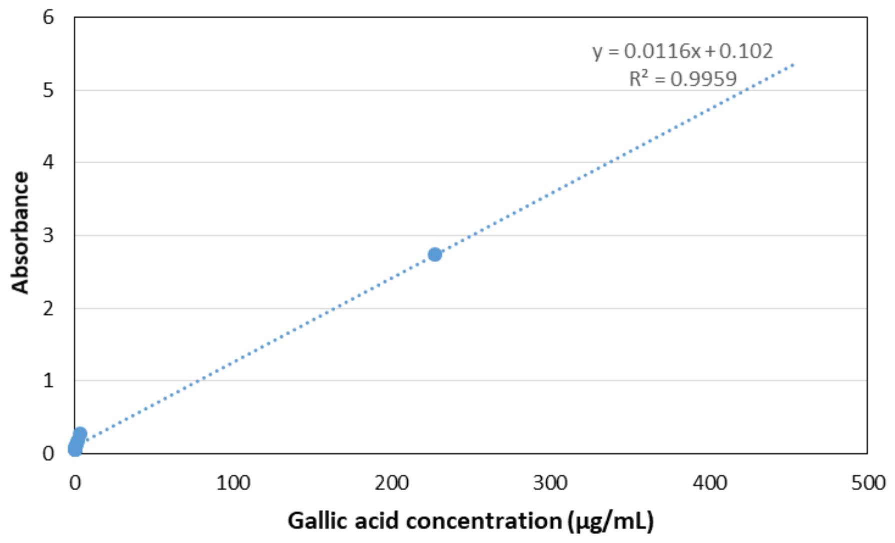

Figure A3: Standard Curve Total Flavanoid Content of Quercetin

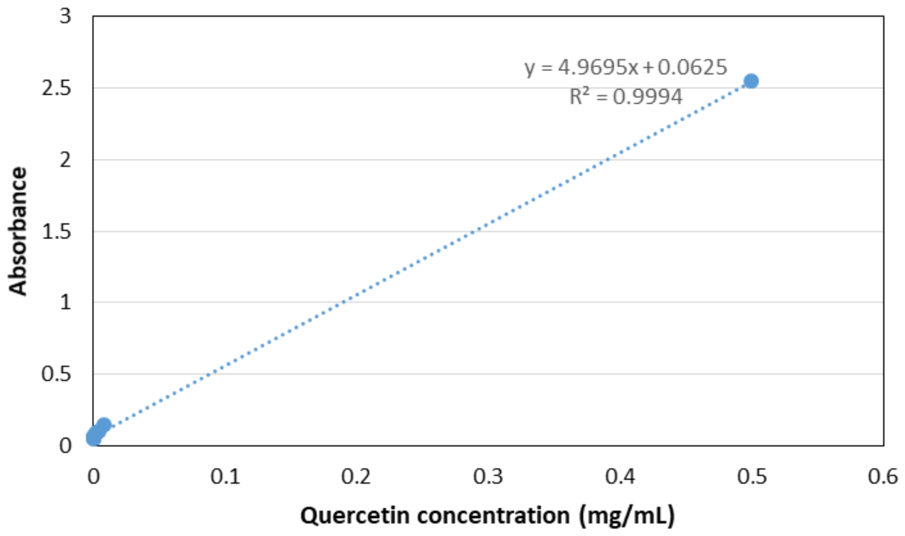

Supplement: Supplementary file 1 [file antioxidants-08-00039-s001.pdf]
